# Mood Monitoring, Mood Tracking, and Ambulatory Assessment Interventions in Depression and Bipolar Disorder: Systematic Review and Meta-Analysis of Randomized Controlled Trials

**DOI:** 10.2196/84020

**Published:** 2026-01-07

**Authors:** Laurence Astill Wright, Georgina Shajan, Daljit Purewal, Jonathan Stone, Madiha Majid, Boliang Guo, Richard Morriss

**Affiliations:** 1 Institute of Mental Health, University of Nottingham, Jubilee Campus, Triumph Road, Nottingham, NG7 2TU, United Kingdom, 44 0115 823 1294; 2 Centre for Academic Mental Health, Population Health Sciences, University of Bristol, Bristol, United Kingdom; 3 Avon and Wiltshire Mental Health Partnership NHS Trust, Bristol, United Kingdom; 4 Coventry and Warwickshire Partnership NHS Trust, Coventry, United Kingdom; 5 NIHR ARC East Midlands, University of Nottingham, Nottingham, United Kingdom; 6 Nottingham NIHR Biomedical Research Centre, University of Nottingham, Nottingham, United Kingdom; 7 NIHR MindTech Medical Technology Collaborative, University of Nottingham, Nottingham, United Kingdom

**Keywords:** bipolar, depression, ecological momentary assessment, mood-tracking, mood-monitoring, self-monitoring

## Abstract

**Background:**

Mood monitoring is widely used by people with depression and bipolar disorder (BD) to prevent relapse and improve insight into their condition, but it is unclear if these interventions have an impact on symptoms and for whom. As the capacity for passive mood monitoring increases, it is vital to improve our understanding of frequent mood assessment.

**Objective:**

This systematic review and meta-analysis assessed the effect of mood monitoring interventions in people with depression and BD to decrease relapse risk and symptoms of depression and mania.

**Methods:**

We conducted a systematic review and meta-analysis (PROSPERO, International Prospective Register of Systematic Reviews: CRD42023396473) and reported results according to PRISMA (Preferred Reporting Items for Systematic Reviews and Meta-Analysis) guidelines. Randomized controlled trials with clinically important follow-up periods were identified via multiple database searches and rated for risk of bias using the Cochrane Risk of Bias tool. The primary outcomes were symptoms of depression and mania. Available data were pooled to calculate standardized mean differences (SMDs) for the primary outcomes: severity of depression, bipolar depression, and mania/hypomania.

**Results:**

We included 8 trials of 1230 participants and 6 different mood monitoring protocols. In BD, meta-analysis found a small but not statistically significant effect of mood monitoring interventions on decreasing mania symptoms (6 comparisons, n=873; SMD 0.16, 95% CI−0.34 to 0.01; *P*=.06) and no effect on bipolar depression (6 comparisons, n=873; SMD −0.08, 95% CI −0.31 to 0.15; *P*=.02). In depression, we found a small effect in decreasing symptoms of depression of borderline statistical significance at 12 months (2 comparisons, n=262; SMD −0.25, 95% CI −0.49 to 0.00; *P*=.05) but not at 6 months (2 comparisons, n=268; SMD −0.21, 95% CI −0.54 to 0.12; *P*=.21). There was an absence of evidence on the effect of mood monitoring on decreased relapse rates or readmission rates. Studies had a low risk of bias. There was no evidence on mood monitoring through ecological momentary assessment.

**Conclusions:**

Overall mood monitoring interventions do not increase or decrease mood symptoms in people with BD, nor is there robust evidence of such effects in people with unipolar depression. Further research is merited on different forms of mood monitoring and to determine under what circumstances mood monitoring might have beneficial or adverse effects. These results initially suggest that ambulatory assessment does not induce large placebo effects or significantly negatively or positively affect mood, and thus that mood monitoring may be an appropriate outcome measure for research or for clinical practice.

## Introduction

Many people with bipolar disorder (BD) and depression track their mood symptoms over time, and there are multiple tools available freely on the web to do this [1-4]. A recent survey of people with BD found that 41.6% of participants reported using a self-management app related to mood or sleep [5,6]. Mood monitoring is widely used, specifically by people with mood disorders [7], for example, Bipolar UK’s mood tracking app has greater than 10,000 downloads on Android alone [8]. Mood tracking is one of the most common features of mental health smartphone apps—previous reviews have noted that mood/behavior tracking is present in over half of these apps [9-11]. Many smartphones include in-built self-tracking functions for health [12], and many studies are incorporating mood monitoring as a method of clinical outcome [13] and testing them as interventions [14].

Traditionally, mood monitoring is done place using paper-based charting [15]. However, many people with BD and depression prefer digital methods as they are convenient and store easily accessible records of mood, allowing people to more easily look back and identify patterns of improvement or worsening of symptoms [8]. Digital methods, where individuals record their mood in the moment, may also decrease recall bias, so there might be greater accuracy in charting and plotting mood rather than retrospective completion of data every few weeks [7]. The technology used in mood tracking and ambulatory assessment is wide-ranging, and some of the descriptive terms used throughout this paper are nuanced, often with overlapping definitions. Because of this, we have listed these important terms in Table 1 below.

**Table 1.** Definitions of forms of mood-monitoring and related terms.

Term	Definition
Self-monitoring	The appraisal and recording of one’s current state, can include mood.
Mood tracking/mood monitoring	Regular recording of one’s mood over a period of time. This can be done digitally on a device or analog using pen and paper charting.
Ambulatory assessment	Wide group of digital methods recording data on the user in real time and in natural settings. Includes mood tracking/monitoring, remote measurement technology, and ecological momentary assessment.
Active data collection	Users input information about their own current state.
Passive data collection	Behavioral data is automatically recorded via technology.
Remote measurement technology	Wearable devices record passive data.
Ecological momentary assessment	Intensive “in the moment” self-reporting by the user, for example, multiple times per day.

Mood monitoring can be used as an intervention (both in randomized controlled trials [RCTs] and nonrandomized studies) and also as a method of ascertaining outcome (both in RCTs and nonrandomized studies). Passive data collection may reduce the burden of data completion and remind the participant less frequently about their mood [16,17]. Some mood monitoring may combine active and passive monitoring. For example, passive monitoring of certain activity or behavior may trigger active data collection from the user when there is a preset level of change in this activity/behavior. In depression, activity may be reduced, and in mania, it may increase [18]. Other forms of mood monitoring might randomly request the participant to actively complete data on mood without any passive monitoring [17]. These technological advances may provide new utility to a relatively old intervention methodology. However, there is a need to assess whether these newer approaches to mood monitoring have benefits or harms as well, and so we included all of these approaches in this review.

There is evidence that increasing awareness of mood fluctuations can improve insight, and the identification of early warning signs can prevent relapse in depression and BD [19]. This raises the question of whether mood tracking can have any direct clinical effects, either positive or negative [20]. Currently, it is unclear if mood monitoring or mood tracking as an intervention is effective in reducing symptom severity or in preventing relapse. It is also possible that mood monitoring interventions have negative effects on mood [21,22] or lead to a response bias whereby users complete the same score despite mood altering in response to being asked the same questions repeatedly. As the capacity for mood monitoring through digital assessment increases and these methods are used increasingly as assessment methods in research, it is vital to improve our understanding of frequent mood assessment [23].

Some people with BD report that mood monitoring helps them to reduce relapse risk, for example, through greater awareness of their current mental state, while others report it worsens their mood, for example, by reminding them of their mood problems, so they consider mood monitoring a burden [16,22]. Others report that it is relatively simple to carry out in their day-to-day lives [1]. Mood monitoring may represent an intervention that is usable, acceptable, and easy for individuals to implement in their lives, and it can be coupled quite easily with simple psychological interventions such as psychoeducation [24]. Questions remain, however, about definitive efficacy and potential for harm from mood monitoring alone.

The potential for efficacy or adverse events is particularly important as RCTs and observational studies increasingly move towards digital combinations of passive and active monitoring or ambulatory assessment outcomes [17,25]. It is thus integral to know whether the method of assessment itself may carry any therapeutic benefits or any adverse effects. Any such risk or benefit might bias any outcome assessment of other interventions in trials, potentially enhancing or obscuring any true benefit or suggesting a false benefit through measurement methods used in the trial rather than from the intervention itself [26]. Furthermore, if there were adverse effects, ambulatory assessment protocols would need to consider providing coping/mitigating strategies—currently, the risk is not known, and such mitigating strategies are not routinely provided [27,28]. The risk/benefit of mood monitoring might be best investigated through an analysis of RCTs of frequent mood assessment, although other approaches, such as reports of adverse events, qualitative monitoring, and surveys from practice, all have their place in responsible technological innovation [29].

Previous reviews have explored mood monitoring but have not assessed efficacy in high-quality RCTs [28,30,31]. This is the first systematic review and meta-analysis that we are aware of that examines mood monitoring as an intervention in RCTs in BD. The aim of this systematic review is to assess the effect of mood tracking in people with BD and depression on relapse risk and symptoms of bipolar depression, mania/hypomania, and depression.

## Methods

### Overview

We used the Cochrane Handbook for Systematic Reviews of Interventions methodology and used a Preferred Reporting Items for Systematic Reviews and Meta-Analysis (PRISMA) checklist. The study was preregistered with the International Prospective Register of Systematic Reviews (PROSPERO: CRD42023396473 [32]).

### Inclusion Criteria

The inclusion criteria were as follows: self-monitoring/ecological momentary assessment (EMA)/repeated symptom assessment in people with BD or depression as an intervention over a minimum period of 3 months, with rating of symptoms weekly at a minimum. On discussion with patient and public involvement, mood monitoring over periods less than 3 months might be misleading in various contexts associated with temporary change, such as changes in medication, menstrual, seasonal, or life event effects on mood, or as the person becomes familiar with a new method of mood monitoring. The studies needed to use an appropriate nonmood monitoring/EMA control. The studies should either use a validated measure of mood or validate the chosen measure with a validated mood measure. The studies could be published in any language and could be digital or nondigital, although we acknowledged that the majority of studies would use digital technologies. We only included RCTs with 20 or more participants with BD or depression [33]. We did not exclude RCT studies where mood monitoring was included but not the primary focus of the intervention (but it so happened that in the included studies, the mood monitoring did comprise a significant part of the overall intervention). We searched the gray literature (eg, conference abstracts, dissertations, policy literature, reports via ProQuest and Google Scholar—full details below) for unpublished studies that were eligible for inclusion.

### Search Strategy and Selection Criteria

The complete search strategy is listed in the Multimedia Appendix 1. We searched PsychINFO, EMBASE, SCOPUS, IEEE Xplore, Ovid MEDLINE, Proquest SciTech Collection, ProQuest Dissertations and Theses Global, and Google Scholar using the search terms. Search results were exported for appraisal and stored on Rayyan [34]. The initial search was conducted on March 03, 2023, and updated on October 28, 2024. All abstracts were appraised by 2 independent screeners (LAW, GM, GS, RP, MM, and DP), and any disagreements were discussed, and a consensus arrived upon, with adjudication by a third independent screener if required. The full text of any potentially relevant papers was acquired, and if we were unable to source the full text of the study, we then contacted the corresponding author to request the paper. To determine if potentially relevant studies met the inclusion criteria, the full text was reviewed separately by 2 authors, again with discussion and consensus with a third reviewer if necessary. All papers for inclusion were reference checked along with relevant systematic reviews [15,18,27,28,30,31,35-38]. Key authors were also emailed to see if the inclusion of any ongoing unpublished studies could be included.

### Data Extraction

Two independent reviewers extracted data (as per symptom severity scores for different time points) from studies meeting the inclusion criteria using identical data extraction forms. Irregularities in the data extraction were discussed, and any discrepancies were resolved through discussion.

### Assessment of Study Bias

The Cochrane Collaboration’s Risk of Bias 2 tool was used for each study [39]. Risk of bias was assessed by 2 independent reviewers (LAW and GS), and any disagreement was resolved via discussion.

The certainty of the evidence for the meta-analysis results for each outcome was assessed independently and in duplicate by 2 review authors (LAW and GS) using the Grading of Recommendations, Assessment, Development, and Evaluation framework. This involved an individual assessment of each of the 5 domains of risk of bias (inconsistency, indirectness, imprecision, and publication bias), resulting in an overall assessment of the certainty of the evidence as “high,” “moderate,” “low,” or “very low” [40].

### Synthesis of Results

The primary outcome in the meta-analysis for treatment studies was a reduction in depression/mania/hypomania incidence/symptoms for people with BD, and a reduction in depressive symptoms at 6 months postintervention. This timeframe was chosen to demonstrate the stability of treatment effects. For mania/hypomania/depression severity, we calculated standardized mean differences (SMDs). Insufficient studies examined BD or depression incidence or relapse risk to meta-analyze.

For outcomes included in more than one study, we measured statistical heterogeneity by calculating the I^2^ statistic [41]. An I^2^ of less than 30% was taken to indicate mild heterogeneity, and a fixed-effects model was used. When the I^2^ was greater than or equal to 30%, a random-effects model was used. All analyses were performed using Review Manager (version 5.3; Cochrane Collaboration).

## Results

### Overview

**Figure 1.**
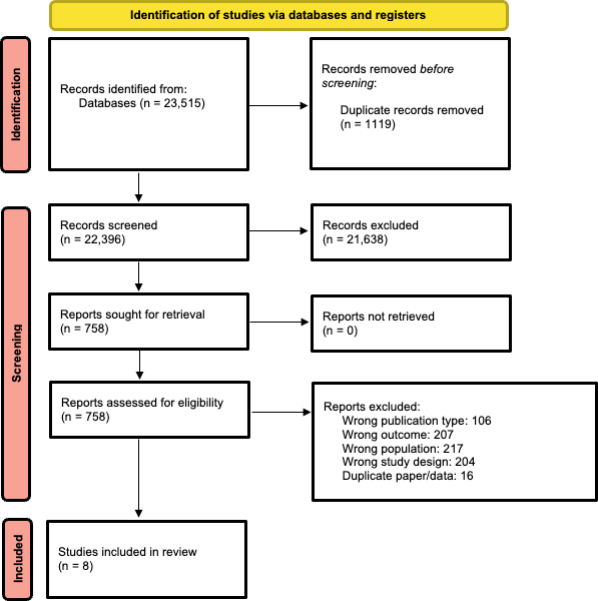
PRISMA (Preferred Reporting Items for Systematic Reviews and Meta-Analysis) flowchart.

The search identified a total of 23,515 studies. No studies that were not in English were found to meet the inclusion criteria. Following title and abstract screening, 21,638 studies were excluded, resulting in a total of 758 studies being reviewed in full. A total of 5 trials in people with BD and 3 trials in people with depression met the eligibility criteria and were included in the review. The BD trials included 803 participants, and the depression trials included 427 participants. Tables 2-4 display detailed characteristics of the studies and the mood monitoring protocols used. Figure 1 and Checklist 1 detail the search strategy with the PRISMA flowchart and checklist.

**Table 2.** Characteristics of included bipolar disorder studies.

Study	Country	Sample^a^	Age (years), mean (SD)	Female (%)^a^	Intervention	Comparator	Setting	Mood monitoring intervention	Active or passive mood monitoring	Mood monitoring duration (months)	Primary outcome
Faurholt-Jepsen et al [42] (n=67)	Denmark	Bipolar 1: 67%, Bipolar 2: 33%	29.3 (8.43)	67	MONARCA system plus: (1) Study nurse reviewing data and contacting patients if signs of deterioration to offer advice. (2) Self-monitored data graphically visualized.	Normal smartphone use	Secondary care: specialist mood disorder service for patients with a new diagnosis of bipolar or treatment resistance.	Daily smartphone self-monitoring: mood, sleep duration, medication taken, activity, irritability, mixed mood, cognitive problems, alcohol consumption, stress, menstruation, individualized EWS^b^.	Active and passive	6	HDRS^c^ and YMRS^d^ at 1‐6 months
Faurholt-Jepsen et al [43] (n=129)	Denmark	Bipolar 1: 59%, Bipolar 2: 41%	43 (12)	59	Monsenso system plus: (1) Study nurse reviewing data and contacting patients if signs of deterioration to offer advice. (2) self-monitored data graphically visualized.	Normal smartphone use	Secondary care: specialist mood disorder service for patients with a new diagnosis of bipolar or treatment resistance	Daily smartphone self-monitoring items: mood, sleep duration, medication taken, activity, irritability, mixed mood, cognitive problems, alcohol consumption, stress, menstruation, individualized EWS, anxiety, self-defined personal parameters, free-text note. Objective smartphone data: phone usage, social activity, step count, GPS location.	Active and passive	9	HDRS and YMRS at 1‐9 months
Faurholt-Jepsen et al [44] (n=98)	Denmark	Bipolar 1: 58%, Bipolar 2: 42%	42.69 (13.46)	52	Monsenso system plus: (1) study nurse reviewing data and contacting patients if signs of deterioration to offer advice. (2) self-monitored data graphically visualized.	Usual care	Secondary care: specialist mood disorder service for patients with a new diagnosis of bipolar or treatment resistance.	Daily smartphone self-monitoring items: mood, sleep duration, medication taken, activity, irritability, mixed mood, cognitive problems, alcohol consumption, stress, menstruation, individualized EWS, anxiety, self-defined personal parameters, free-text note. Objective smartphone data: phone usage, social activity, step count, GPS location.	Active and passive	6	Rate and duration of psychiatric readmissions at 3‐6 months
Gliddon et al [45] (n=304)	Australia and the United States	Bipolar 1: 55%, Bipolar 2: 38%	39.47 (11.19)	82	Intervention 1: Discussion forum plus MoodSwings-Plus: MoodSwings plus additional CBT-based^e^ interactive elements: tools to support mood and medication monitoring, life-chart development, cognitive strategies, motivational interviewing techniques, self-reflection, problem solving, identification of personal triggers and a relapse prevention plan. Intervention 2: Discussion forum plus MoodSwings: Online intervention comprising mood monitoring, assessing prodromal mood states, preventing relapse, and setting SMART goals. Online delivery of MAPS (Mood Assessment Prevent SMART) program.	Discussion forum	Mixed sample: participants recruited via advertising.	Online mood monitoring via MoodSwings and MoodSwings-Plus websites.	Active	12	MADRS^f^ and YMRS at 3‐12 months
Goulding et al [14] (n=205)	The United States	Bipolar 1: 100%, Bipolar 2: 0%	42 (12)	61	Livewell	Usual care	Secondary care: 1 previous mood episode in the past year and current care by psychiatrist/nurse practitioner.	Smartphone-based self-management intervention: daily and weekly check-ins for weeks 1‐16. Daily: adherence, sleep, duration, routine, wellness levels. Weekly: symptom severity scoring for all individual DSM-IV^g^ mood symptoms.	Active	4	Time to relapse

a Reference articles do not provide the complete statistics, so absolute values corresponding to percentage values cannot be provided here.

b EWS: Early Warning Signs.

c HDRS: Hamilton Depression Rating Scale 17 items.

d YMRS: Young Mania Rating Scale.

e CBT: cognitive behavior therapy.

f MADRS: Montgomery-Åsberg Depression Rating Scale.

g DSM: Diagnostic and Statistical Manual of Mental Disorders.

**Table 3.** Intervention protocols of included studies for depression and bipolar disorder.

Study type and respective included studies	Mood monitoring intervention	Mood monitoring duration (months)	Active or passive mood monitoring	Analog or digital mood monitoring	Adherence to mood monitoring	Trial attrition
Included studies for bipolar disorder						
Faurholt-Jepsen et al [42]	Daily smartphone self-monitoring: mood, sleep duration, medication taken, activity, irritability, mixed mood, cognitive problems, alcohol consumption, stress, menstruation, individualized EWS^a^, clinical feedback loop.	6	Active and passive	Digital	>93% of patients randomized to the intervention group self-reported on a daily basis.	Intervention group: 3% attrition over 6 months, control group: 3% attrition over 6 months.
Faurholt-Jepsen et al [43]	Daily smartphone self-monitoring items: mood, sleep duration, medication taken, activity, irritability, mixed mood, cognitive problems, alcohol consumption, stress, menstruation, individualized EWS, anxiety, self-defined personal parameters, free-text note. Objective smartphone data: phone usage, social activity, step count, GPS location, clinical feedback loop.	9	Active and passive	Digital	Over 9 months, patients in the intervention group adhered to the daily self-monitoring 72.6% of the days.	Intervention group: 7% attrition at 9 months, control group: 7% attrition at 9 months.
Faurholt-Jepsen et al [44]	Daily smartphone self-monitoring items: mood, sleep duration, medication taken, activity, irritability, mixed mood, cognitive problems, alcohol consumption, stress, menstruation, individualized EWS, anxiety, self-defined personal parameters, free-text note. Objective smartphone data: phone usage, social activity, step count, GPS location, clinical feedback loop.	6	Active and passive	Digital	80.6% adherence to daily self-monitoring in intervention group over 6 months.	Total attrition: 35% at 6 months, intervention group: 22%, control group: 53%.
Gliddon et al [45]	Intervention 1: Discussion forum plus MoodSwings-Plus: MoodSwings plus additional CBT-based^b^ interactive elements—tools to support mood and medication monitoring, life-chart development, cognitive strategies, motivational interviewing techniques, self-reflection, problem solving, identification of personal triggers, and a relapse prevention plan. Intervention 2: Discussion forum plus MoodSwings: Online intervention comprising mood monitoring, assessing prodromal mood states, preventing relapse, and setting SMART goals. Online delivery of MAPS (Mood Assessment Prevent SMART) program.	12	Active	Digital	Control group: 89% accessed the discussion forum, MoodSwings group: 86% accessed the modules, MoodSwings-Plus: 74% accessed the tools.	Total attrition: 9% at 12 months, control group: 6%, MoodSwings group: 7%, MoodSwings-Plus: 13%.
Goulding et al [14]	Smartphone-based self-management intervention: daily and weekly check-ins for weeks 1‐16. Daily: adherence, sleep, duration, routine, wellness levels. Weekly: symptom severity scoring for all individual DSM-IV^c^ mood symptoms.	4	Active	Digital	The mean (SE) percentage of daily check-ins completed during weeks 1 through 4 was 78% (3%), 74% (3%), 71% (3%), and 64% (3%), respectively, 66% (3%) during week 6, and 47% (4%) during week 16.	Intervention group: 15% attrition at 4 months, control group: 15% attrition at 4 months.
Included studies for depression						
Aikens et al [46]	Automated Interactive Voice Response telephone calls assessing symptom severity: PHQ-9^d^ and antidepressant adherence.	12	Active	Analog—telephone	22 % in intervention arm completed<50% of scheduled calls	Total attrition: 14%, intervention: 17%, control: 10%.
Tonning et al [47]	Monsenso system plus: (1) Study nurse reviewing data and contacting patients if signs of deterioration to offer advice, (2) self-monitored data graphically visualized, and (3) smartphone-based CBT modules	6	Active and passive	Digital	82.7% in intervention arm	Total attrition: 82.5%, intervention: 20%, control: 15%.
Hunkeler et al [48]	Personalized self-monitoring via eCare for Moods—tracking health-related disability, medication adherence, side effects, alcohol and drug use, new symptoms, early warning signs. Graphs of monitoring data displayed over time.	24	Active	Digital	≈87% entered any monitoring data over the first 6 months, ≈45 % entered any monitoring data over the second 6 months	Total attrition: 16%, intervention attrition: 22%, control attrition: 12%.

a EWS: early warning signs.

b CBT: cognitive behavior therapy.

c DSM: Diagnostic and Statistical Manual of Mental Disorders.

d PHQ-9: Patient Health Questionnaire-9.

**Table 4.** Characteristics of included depression studies.

Study	Country	n	Age (years), mean (SD)	Female (%)^a^	Intervention	Comparator	Setting	Mood monitoring intervention	Active or passive mood monitoring	Mood monitoring duration	Primary outcome
Aikens et al [46]	The United States	204	49^b^	81	Automated Interactive Voice Response telephone calls	Enhanced usual care: usual care plus printed self-management material at baseline and assigned family/friend to discuss this with weekly	Primary care	Automated Interactive Voice Response telephone calls assessing symptom severity—PHQ-9^c^ and antidepressant adherence.	Active	12 months	PHQ-9 at 6‐12 months
Tonning et al [47]	Denmark	120	Intervention: 44.5 (14.0), Control: 43.4 (14.3)	Intervention: 47.5 (28), Control: 43.4 (14.3)	Monsenso system plus: 1. study nurse reviewing data and contacting patients if sign of deterioration to offer advice 2. self-monitored data graphically visualized 3. smartphone-based CBT^d^ modules	TAU^e^	Tertiary care: specialist mood disorder service for patients with a new diagnosis of bipolar or treatment resistance	Daily smartphone self-monitoring items: mood, sleep duration, medication taken, activity, irritability, mixed mood, cognitive problems, alcohol consumption, stress, menstruation, individualized EWS^f^, anxiety, self-defined personal parameters, free-text note. Objective smartphone data: phone usage, social activity, step count, GPS location	Active and passive	6 months	Rate and accumulated duration of psychiatric readmissions. (HDRS-17^g^ as secondary outcome)
Hunkeler et al [48]	The United States	103	Intervention: 48.49 (12.83), Usual care: 51.88 (10.56)	79.6	eCare for moods: website offering personalized self-monitoring, messaging with eCare manager, depression psychoeducation, CBT modules, online discussion group, problem-specific advice, personal database, task lists, appointment calendar.	TAU	Secondary care	Personalized self-monitoring via eCare for moods: tracking health-related disability, medication adherence, side effects, alcohol and drug use, new symptoms, early warning signs. Graphs of monitoring data displayed over time.	Active	12 months	Psychiatric Status Rating for Depression of 6 questions adapted from SCID^h^ measured weekly

a Reference articles do not provide the complete statistics, so absolute values corresponding to percentage values cannot be provided here.

b Reference articles do not provide the complete statistics, so the SD value cannot be provided alongside mean value cannot be provided here.

c PHQ-9: Patient Health Questionnaire-9.

d CBT: cognitive behavior therapy.

e TAU: treatment as usual.

f EWS: Early Warning Signs.

g HDRS-17: Hamilton Depression Rating Scale 17 items.

h SCID: Structured Clinical Interview for DSM-IV.

### Bipolar Disorder: Overview of Individual Study Findings

In BD, 5 trials [14,43-45,49-61] used 3 different mood monitoring protocols. These were the MONARCA/Monsenso system, Livewell, and MoodSwings [45,52]. Only the MONARCA/Monsenso system incorporated passive data capture into the intervention. These were all digital; 3 used active and passive ambulatory assessment [43,44,51] while 2 just used active ambulatory assessment [14,45].

All studies assessed mania and depression severity using a mixture of self-report and clinical rating scales, with three of these as the primary outcome [43,45,62]. Two studies assessed relapse rate/psychiatric readmission or duration as the primary outcome [44]. Only one study [45] provided raw mania/depression severity data, and we contacted the authors of the other papers to obtain this for the analyses. Studies used similar inclusion criteria, but there were some key differences. All studies recruited individuals with BD from clinical services and confirmed BD via a structured clinical interview at baseline and then relied on self-report measures/clinical ratings for outcome assessment. All studies excluded individuals who were currently experiencing a major mood episode, either by using a cutoff on self-report scores/clinical rating scales (which varied between studies) [14,42,45] or by the participant completing treatment at a specialist mood disorder service [43], with one study recruiting individuals on discharge from inpatient care following hospitalization for an affective episode [44].

The results of the trials were mixed. The 2 trials [14,47] assessing relapse/readmission risk showed no effect of mood monitoring. Goulding et al [14] demonstrated an effect of decreased relapse risk for low-risk individuals, but no effect on percentage-time symptomatic for all participants. Three studies did not identify a decrease in depression or mania scores from mood monitoring on clinical ratings conducted blinded to allocation [43,51,63]. Gliddon et al [45] reported decreases in depressive symptoms when compared with the peer support control, Goulding et al [14] also reported improvements in depression severity alongside improved relational quality of life.

Faurholt-Jepsen et al [51] and Faurholt-Jepsen et al [43] found a nonstatistically significant trend for worsening depressive symptoms versus the control group.

### Bipolar Disorder: Meta-Analysis

**Figure 2.**
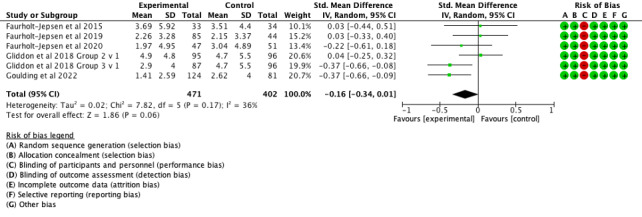
Forest plot of effects of mood monitoring interventions for the treatment of symptoms of mania/hypomania in people with BD at 6‐12 months [14,42-45]. BD: bipolar disorder.

**Figure 3.**
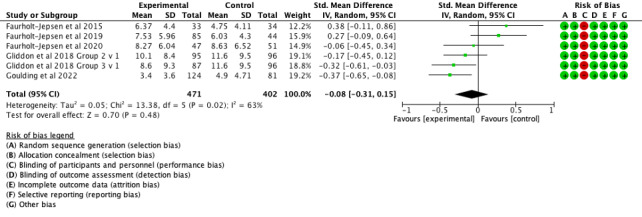
Forest plot of effects of mood monitoring interventions for the treatment of symptoms of depression in people with BD at 6‐12 months [14,42-45].

Concerning the primary outcome, there was no effect of mood monitoring interventions in reducing symptoms of mania/hypomania (Figure 2: 6 comparisons, n=873; SMD −0.16, 95% CI −0.34 to 0.01; *P*=.06; I^2^=36%) or bipolar depression (Figure 3: 6 comparisons, n=873; SMD −0.08, 95% CI −0.31 to 0.15; *P*=.02; I^2^=63%).

### Depression: Overview of Individual Study Findings

In depression, 3 trials used 3 different mood tracking procedures. These were: interactive voice response telephone calls (IVR) [46], Monsenso [47], and eCare for Moods [48]. None of the trials used mood monitoring as the primary outcome; instead, using standardized infrequent assessments of mood (Tables2  4). One study was analog using the telephone [46], the other two were digital [47,48]; two used active ambulatory assessment [48], with one using active and passive ambulatory assessment [47].

The Monsenso [47] and eCare for Moods [48] protocols used daily monitoring, while IVR calls [46] were administered weekly. Only the Monsenso system incorporated passive data collection. All protocols incorporated clinical feedback of the assessment as an intervention. Monseno and eCare for Moods used digital technology, while IVR used telephone calls and voice messages. We did not identify any paper-based charting trials. The duration of ambulatory assessment protocols varied from 6 months to 24 months.

Two studies assessed relapse rate/psychiatric readmission or duration as the primary outcome [46,48]. Tonning et al [64] assessed the rate and accumulated duration of psychiatric readmissions as the primary outcome, assessing depression severity as a secondary outcome. Only one study [48] provided raw depression severity data, and we contacted the authors of the other papers to obtain this for the analyses. Studies used similar inclusion criteria, but there were some key differences. All studies recruited individuals with depression from clinical services. Two studies confirmed depression via a structured clinical interview at baseline, while Aikens et al [46] confirmed depression via the Patient Health Questionnaire-9. All studies then relied on self-report measures for outcome assessment. Aikens et al [46] excluded patients who were experiencing major psychiatric distress and recruited from community samples. Hunkeler et al [48] did not exclude individuals with suicidal ideation or a particular severity of depression, again recruiting from community clinics. Tonning et al [47] recruited people with depression receiving inpatient care, providing the intervention postdischarge.

The results of the 3 trials were mixed. Tonning et al [47] report no change in relapse risk or readmission duration, as well as no change in depressive symptoms. They did, however, report a range of benefits across tertiary outcomes when adjusted for age, sex, and Hamilton Depression Rating Scale scores. Patients in the intervention group reported statistically higher recovery, measured using the Recovery Assessment Scale, as well as a tendency (not statistically significant) towards higher quality of life, higher well-being, more satisfaction with treatment, and higher behavioral activation in the intervention group compared with the control group.

In eCareformoods, participants in the intervention group experienced a statistically significant reduction in depressive symptoms at 2 years. A higher proportion of those in the intervention group remained in recovery from their depression, and the number needed to treat was calculated at 8. Intervention participants also had improvements across a range of secondary outcomes, including improved general mental health, learning new coping skills, greater satisfaction with specialty care, and more confidence in managing depression. These were all statistically significant.

Aikens et al [46] report a statistically significant improvement in Patient Health Questionnaire-9 depression severity (2.5 points) at 6 months in the intervention group. This persisted for 12 months. Clinical response was more likely in the intervention group than the control at 6 months, but this difference decreased in size and lost statistical significance by 12 months.

Only Tonning et al [47] reported adverse effects, and these were as follows: 3 participants found the monitoring stressful, and 1 participant did not find it helpful.

### Unipolar Depression: Meta-Analysis

**Figure 4.**
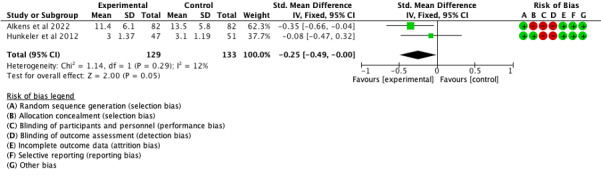
Forest plot of effects of mood monitoring interventions in reducing symptoms of depression at 12 months [46,48].

**Figure 5.**
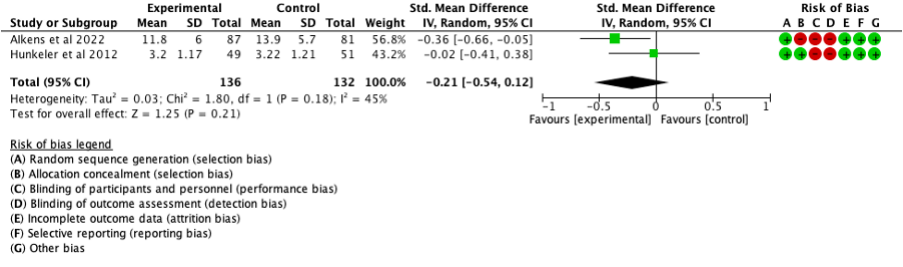
Forest plot of effects of mood monitoring interventions in reducing symptoms of depression at 6 months [46,48].

There was a small effect of borderline statistical significance at 12 months (Figure 4: 2 comparisons, n=262; SMD −0.25, 95% CI −0.49 to 0.00*; P*=.05; I^2^=45%) but not at 6 months (Figure 5: 2 comparisons, n=268; SMD −0.21, 95% CI −0.54 to 0.12; *P*=.21; I^2^=12%). Only 2 trials were included in the meta-analysis as the appropriate data for Tonning et al [47] were not available.

### Risk of Bias Assessments

The quality of these RCTs was good, with all trials having low risk of bias (Table 5). All trials used intention-to-treat analysis. All studies reported adherence and attrition.

**Table 5.** Risk of bias assessments for included bipolar disorder and depression studies.

Study	Risk of bias criteria							
	Random sequence generation	Allocation concealment	Blinding of participants and personnel	Blinding of outcome assessment	Incomplete outcome data	Selective reporting	Other sources of bias	Total number of low-risk domains
Risk of bias assessments for included bipolar disorder trials								
Faurholt-Jepsen et al [42]	Low risk	Low risk	High risk	Unclear	Low risk	Low risk	Low risk	6
Faurholt-Jepsen et al [43]	Low risk	Low risk	High risk	Low risk	Low risk	Low risk	Low risk	6
Faurholt-Jepsen et al [44]	Low risk	Low risk	High risk	Low risk	Low risk	Low risk	Low risk	6
Gliddon et al [45]	Low risk	Low risk	High risk	Low risk	Low risk	Low risk	Low risk	6
Goulding et al [14]	Low risk	Low risk	High risk	Low risk	Low risk	Low risk	Low risk	6
Risk of bias assessments for included depression trials								
Aikens et al [46]	Low risk	Low risk	High risk	High risk	Low risk	Low risk	Low risk	5
Tonning et al [47]	Low risk	Low risk	High risk	Low risk	Low risk	Low risk	Low risk	6
Hunkeler et al [48]	Low risk	Low risk	High risk	High risk	Low risk	Low risk	Low risk	5

## Discussion

### Principal Findings

This systematic review of mood monitoring interventions in people with BD found no effect either way on symptoms of mania/hypomania or bipolar depression in people with BD at 6‐12 months. There was no robust evidence of mood monitoring either increasing or decreasing symptoms of depression, with no effect at 6 months and borderline statistical improvement at 12 months in only 2 RCTs. There were some other benefits of mood monitoring across 3 RCTs in depression, but there was no consistency in what was measured or the outcomes that were improved.

### Bipolar Disorder

Mood monitoring is theorized to work by improving understanding and insight to enable people to self-manage their BD [16]. People with BD seem to use the data provided to them by mood monitoring in varied ways [65]. These appear to be highly personalized and tailored to what works best for them. The mood monitoring protocol provides a platform for people to interpret their own mood data, devising highly personal ways of self-managing their BD subsequently [66]. This self-management may focus on sleep, medication, crisis planning, and communication in close relationships [16]. Thus, the variability in these outcomes might reflect the different ways in which participants used the mood monitoring information and their coping strategies in the face of depression and mania [8] as well as differences in populations and measurement of mood. Despite the equivocal results reported here, the practice remains hugely popular, and in the digital sphere, there are multiple different apps and protocols available aimed at people with BD, such as the Bipolar UK mood tracker [4], eMoods [42], Moodily, Moodnotes, etc [3].

### Depression

There is some agreement with the findings from the meta-analysis we report from RCTs of mood monitoring in bipolar depression that, on the whole, mood monitoring has neutral effects on outcome, but there is quite a lot of heterogeneity, with some people reporting benefits, and others distress and burden. The findings are not robust. While all 3 RCTs report other benefits of mood monitoring, such as relapse, recovery, increased overall mental health, greater confidence in managing depression, and greater satisfaction with mental health services, there was no consistency in what or how these secondary or tertiary outcomes were measured. Only one of these RCTs reported adverse effects of mood monitoring, with 3 participants finding it a burden and stressful. From qualitative research, some variables that have bearing on the outcome of mood monitoring are context in which mood monitoring is taking place, the usual coping strategies, the nature of mood monitoring, and the population that is being examined. Only one RCT used passive mood monitoring, and none used EMA approaches, so there is no evidence available on modern developments in mood monitoring of depression symptoms in people with unipolar depression. Despite the modest effects reported here, mood monitoring remains popular for people with depression [67-70], and in the digital sphere, there are multiple different apps/protocols available [70]. This review highlights that the popularity of the process may be disproportionate to the direct effects of mood monitoring as an intervention. From the systematic reviews of qualitative data that we have performed, and like self-monitoring of BD, the popularity of mood monitoring in depression may be through the empowerment of the individual to use the data from mood monitoring in a variety of personal ways rather than any direct effect on any outcome [21,22]. However, mood monitoring is not for everyone; for some, it is a burden or a reminder of poor well-being.

### Strengths and Limitations

Our search was thorough and in accordance with Cochrane methodology. We also consulted experts in the field, used a wide search, and used reference searching. Our conclusions, however, were limited due to a paucity of literature. This itself is an important finding, considering the possible implications of frequent mood assessment as ambulatory assessment and EMA approaches develop further. Sometimes there is definitional overlap between EMA protocols and mood monitoring protocols [17]. We did not identify a large enough group of studies using EMA approaches, passive monitoring, or a combination of the two to determine any outcomes or harms from these approaches in this review. We did not include mood monitoring protocols that used shorter follow-up periods, as some previous reviews have done [18-20], because we wanted to examine whether there was any high-quality evidence on benefits or harms. Therefore, we chose RCTs of mood monitoring interventions lasting at least 3 months and clinically relevant follow-up lengths of at least 6‐12 months.

Many other trials were excluded because control groups also had an intervention consisting of some element of mood monitoring. In all the included studies, the main therapeutic intervention applied to all participants in the intervention group was mood monitoring, but all of these trials included additional likely therapeutic elements, which makes isolating the effect of the mood monitoring challenging. With regard to the 5 BD trials, the MONARCA/Monsenseo [43,44,51] studies included data review and outreach by a nurse; the Livewell [14] and Moodswings [45] trials included additional coaching, psychoeducational materials, and planning for management of early warning signs and symptoms. In all 3 RCTs of mood monitoring in depression, there were other elements that might have improved or worsened depression symptoms. Two RCTs used cognitive behavior therapy modules [47,48], and one provided self-management guidance based on the severity of the participant’s symptoms [46]. These additional elements may prove therapeutic benefit but also obfuscate any effect directly from mood monitoring itself. Thus, it may be impossible to determine only the effects of mood monitoring on symptoms of depression or mania because interventions have elements of other interventions as well. There were no studies with just a basic mood monitoring element versus a nonmood monitoring control.

In addition to nonmood monitoring elements of the intervention, there may be factors other than the differences in the intervention that explain some of the mixed results we observe here. These include clinical characteristics of the sample such as the type/subtype of mood disorder (Bipolar 1: Bipolar 2 proportions reported in Tables2  4) and as the results of Goulding et al [14] suggest, there may be improved beneficial effects in lower-risk individuals with BD who are at a specific stage of their illness, and this is supported by qualitative work suggesting that there may be a right time for people with BD to be using mood tracking as an intervention [22,71]. Furthermore, while we did not assess adherence to mood tracking in this paper, we have addressed this in a separate meta-analysis [72], and this separate work demonstrates that all of the studies included here had >70% adherence. Thus, it is unclear if poor adherence levels may cause a failure of effect of mood tracking. Suboptimal adherence may obfuscate these effects, particularly if the effect size is small. However, poor adherence is pragmatic and reflective of real-life outcomes [73], and so these results provide a more pragmatic signal of effect.

Our review focused on the effects of mood monitoring on mood symptoms. However, there might be other benefits from mood monitoring, such as the development of mental literacy about the condition early in its course by seeing how mood varies in severity across time or learning to recognize the symptoms of mania. It may improve confidence or coping strategies that exert some control over the symptoms through developing insights into the conditions. These elements may improve recovery and function [74]. For instance, a valuable role for mood monitoring might be to help with decision-making when seeking help for those who frequently relapse with depression or BD, and when to make important life decisions, such as new responsibilities or decisions with an element of risk, like taking a holiday abroad, away from usual sources of help. Thus, mood monitoring might also have different clinically important outcomes depending on the recency of diagnosis, course of illness, or where recovery and improvement of function are a clinical priority.

### Future Research and Clinical Implications

Future research in mood disorders should evaluate the definitive efficacy of mood monitoring alone rather than additional components to assess for benefits and potential harms. Care should be taken over control groups to ensure that they do not unwittingly include mood monitoring or psychotherapeutic approaches that might obscure the effects of mood monitoring. It remains unclear for whom mood monitoring may be most effective, and future research should assess this. It could be, for example, that it is least effective and most harmful in people with mood disorders who cope with depression by suppressing these symptoms and have not developed other coping strategies. Other groups who may experience harm might be people with mood disorders that feature paranoia or where monitoring of the person was used as a form of coercion, for example, as a feature of morbid jealousy or other abusive relationships. The benefits and harms from more modern approaches to mood monitoring should be explored with a broader range of outcomes focusing on mood monitoring to improve recovery, function, capability, and quality of life.

The field would benefit from a definitive RCT assessing time to relapse in those in asymptomatic remission with an appropriate control group. This would use a mood monitoring intervention with minimal additional psychotherapeutic strategies. This work is important due to the increased use of ambulatory assessment measures as measures of treatment outcomes [75] in studies looking at other interventions and not primarily to explore the effects of measurement itself. These are, in many cases, indistinguishable from mood monitoring protocols that study mood monitoring as an intervention. Such a RCT of mood monitoring might benefit from qualitative work performed alongside to better understand how participants use information gleaned from mood monitoring, as they are not always passive consumers of such information [16]. Finally, we also need to examine mood monitoring in poorer and ethnically diverse populations. These populations may be excluded from digital interventions through poverty or other disadvantages [76], and the characteristics that predispose a population to digital disadvantage are the same as those that might put them at increased risk of mental illness [77,78]. If digital forms of monitoring and interventions are to be used more broadly in health care, they may have the effect of widening pre-existing health inequalities through lack of access to the technologies themselves, as well as to research, in disadvantaged populations. Technology could allow populations who might not otherwise easily access health care to access more relevant information or interventions, in languages other than the one used by the health care provider.

There is insufficient robust, high-quality evidence of benefits or harm to recommend active mood monitoring by participants as primary outcomes in an RCT. In fact, the heterogeneity of outcomes and variability in the way people with mood disorders appraise and use active mood monitoring suggests that active mood monitoring would be unsuitable for use as a primary outcome; a source of variance in outcome might be introduced that would be nonrandom and not necessarily predictable. Since many passive mood monitoring and ambulatory assessments are more implicit measures of mood, participants may not have as much agency in appraising and using such information. However, at this point, these measures are also not suitable as their effects on both outcomes and harms have not been sufficiently tested.

The meta-analysis showed small, nonsignificant, or borderline effects with the use of mood-monitoring, and understanding the practical implications of this is important, particularly considering the popularity of mood-tracking apps, some of which carry recommendations by organizations such as Bipolar UK. Mood monitoring, often coupled with other additional elements in the forms assessed here, did not have clinically important effects, and the qualitative research frequently reporting benefit does not align with the quantitative evidence presented here [21,79]. It is difficult to know under what circumstances mood monitoring may work for these individuals who do report a qualitative benefit. The qualitative research suggests that just mood monitoring is insufficient for any clinical effect and that individuals must incorporate this into a wide range of self-management strategies to keep them well [21,22,79]. The finding that there is no large clinical or placebo effect from mood monitoring suggests that these tools may make excellent outcome measures for research and clinical practice—fundamentally monitoring symptoms over time and using this information as an adjunct to make decisions around care (eg, improvements in shared decision making using more accurate data) and improving existing research outcomes.

### Conclusions

As technological advances are applied to digital health and the capacity for more usable, passive mood monitoring increases, it is vital to understand whether these interventions work and for whom. It is also important to understand any positive or negative effects, as self-tracking is often used as a control or outcome assessment method in studies [17]. For BD, this review showed no effect of mood monitoring on symptoms of mania or bipolar depression, although the evidence was not robust with moderate to high heterogeneity in outcome. In people with depression, there was no robust evidence of the effects of mood monitoring on depression symptoms, with only 2 RCTs contributing to the meta-analysis. There was no evidence of the effect of mood monitoring using EMA. These results initially suggest that ambulatory assessment does not induce large placebo effects or significantly negatively/positively affect mood, and thus that mood monitoring may be an appropriate outcome measure for research or for clinical practice.

## Supplementary material

10.2196/84020 Multimedia Appendix 1 Search strategy.

10.2196/84020 Checklist 1 PRISMA checklist.

## Abbreviations

BD: bipolar disorder
EMA: ecological momentary assessment
IVR: interactive voice response
PRISMA: Preferred Reporting Items for Systematic Reviews and Meta-Analysis
PROSPERO: International Prospective Register of Systematic Reviews
RCT: randomized controlled trial
RCT: randomized controlled trial
SMD: standardized mean difference
